# Supplementary material for: Causal Relationship between Aging and Anorexia Nervosa: A White-Matter-Microstructure-Mediated Mendelian Randomization Analysis
Source: Biomedicines. 2024 Aug 16;12(8):1874. doi: 10.3390/biomedicines12081874 (PMC11351342; doi:10.3390/biomedicines12081874)
Supplement: Supplementary file 1 [file biomedicines-12-01874-s001.zip › Supplementary Figure/Figure S4/Singe SNP plot of MR analysis for TL to ubm-b-1946.pdf]

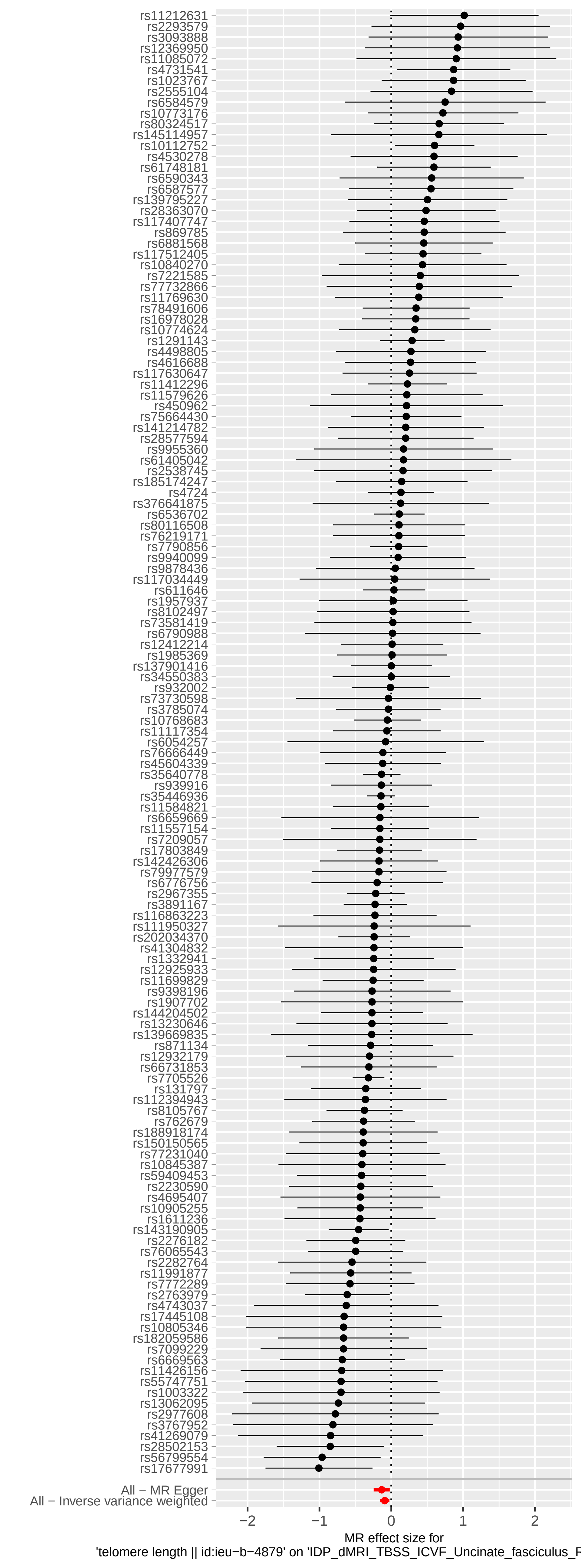
rs11212631
rs2293579
rs3093888
rs12369950
rs11085072
rs4731541
rs1023767
rs2555104
rs6584579
rs10773176
rs80324517
rs145114957
rs10112752
rs4530278
rs61748181
rs6590343
rs6587577
rs139795227
rs28363070
rs117407747
rs869785
rs6881568
rs117512405
rs10840270
rs7221585
rs77732866
rs11769630
rs78491606
rs16978028
rs10774624
rs1291143
rs4498805
rs4616688
rs117630647
rs11412296
rs11579626
rs450962
rs75664430
rs141214782
rs28577594
rs9955360
rs61405042
rs2538745
rs185174247
rs4724
rs376641875
rs6536702
rs80116508
rs76219171
rs7790856
rs9940099
rs9878436
rs117034449
rs611646
rs1957937
rs8102497
rs73581419
rs6790988
rs12412214
rs1985369
rs137901416
rs34550383
rs932002
rs73730598
rs3785074
rs10768683
rs11117354
rs6054257
rs76666449
rs45604339
rs35640778
rs939916
rs35446936
rs11584821
rs6659669
rs11557154
rs7209057
rs17803849
rs142426306
rs79977579
rs6776756
rs2967355
rs3891167
rs116863223
rs111950327
rs202034370
rs41304832
rs1332941
rs12925933
rs11699829
rs9398196
rs1907702
rs144204502
rs13230646
rs139669835
rs871134
rs12932179
rs66731853
rs7705526
rs131797
rs112394943
rs8105767
rs762679
rs188918174
rs150150565
rs77231040
rs10845387
rs59409453
rs2230590
rs4695407
rs10905255
rs1611236
rs143190905
rs2276182
rs76065543
rs2282764
rs11991877
rs7772289
rs2763979
rs4743037
rs17445108
rs10805346
rs182059586
rs7099229
rs6669563
rs11426156
rs55747751
rs1003322
rs13062095
rs2977608
rs3767952
rs41269079
rs28502153
rs56799554
rs17677991
All − MR Egger
All − Inverse variance weighted
−2
−1
0
1
2
MR effect size for
'telomere length || id:ieu-b-4879' on 'IDP_dMRI_TBSS_ICVF_Uncinate_fasciculus_R
